# Long Non-coding RNA CCAT1 Acts as an Oncogene and Promotes Sunitinib Resistance in Renal Cell Carcinoma

**DOI:** 10.3389/fonc.2020.516552

**Published:** 2020-09-25

**Authors:** Liping Shan, Wei Liu, Yunhong Zhan

**Affiliations:** ^1^Department of Urology, Shengjing Hospital, China Medical University, Shenyang, China; ^2^Emergency Department, First Hospital of China Medical University, Shenyang, China

**Keywords:** CCAT1, renal cell carcinoma, sunitinib, resistance, apoptosis

## Abstract

Although sunitinib contributes to prolonging the progression-free survival of metastatic renal cell carcinoma significantly, the universal presence of resistance limits the initial response rate and restricts durable responses. The mechanisms involved in sunitinib resistance vary and need further investigation. We found long non-coding RNA (lncRNA) colon cancer-associated transcript-1 (CCAT1) overexpressed in sunitinib-resistant cells while declined in the parental cells. Moreover, lncRNA CCAT1 increased significantly in samples with resistance to sunitinib compared with those with responses to sunitinib. The reduction of CCAT1 suppressed cell growth and colony formation while triggering apoptosis. Inversely, the ectopic expression of c-Myc reversed the inhibition of cell growth and enhancement of apoptosis by the knockdown of CCAT1. We also verified that anti-apoptosis protein B-cell lymphoma 2 (Bcl-2) and myeloid cell leukemia 1 (Mcl-1) decreased along with the deregulation of CCAT1, whereas the expression of Bcl-2 and Mcl-1 restored in cells that were transfected sh-CCAT1 and c-Myc simultaneously. Apart from the *in vitro* experiments, we demonstrated that knockdown of CCAT1 boosted response to sunitinib by performing sunitinib-resistant ACHN mouse models. Briefly, lncRNA CCAT1 conferred renal cell carcinoma resistance to sunitinib in a c-Myc-dependent manner, providing a novel target for improvement of sunitinib therapy.

## Introduction

Renal cell carcinoma (RCC) implies that cancer emerged in the renal epithelium and attributed to the majority of cancers in the kidney. According to the increasing understanding of the morphological, molecular, and clinical features, the main categories of renal cell carcinomas are clear cell RCC, papillary RCC, and chromophobe RCC, containing 65–70%, 15–20%, and 5% of RCCs, separately (1). Even though localized RCC can be managed well by surgery and radiation therapy, one-third of the limited RCC cases progressed to metastatic disease, which demands systemic therapies. Currently, anti-angiogenic therapy is the first-line treatment for advanced RCC, including bevacizumab, the antibody-targeted vascular endothelial growth factor (VEGF), and tyrosine kinases inhibitor-targeted vascular epidermal growth factor receptors such as sunitinib, pazopanib, and axitinib (2). Nonetheless, anti-angiogenic therapy requires improvement due to a few patients with tumors that have primary resistance and the general appearance of adapted resistance during treatment.

Previous studies have provided several clues for overcoming the resistance to anti-angiogenesis. For instance, the alternative activation of hepatocyte growth factor-mesenchymal-epithelial transition factor axis (HGF-MET axis) drove resistance to VEGF inhibitor (3). The echinoderm microtubule-associated protein-like 4/anaplastic lymphoma kinase (EML4-ALK) fusion genes were implicated in angiogenesis apart from VEGF signaling cascades (4). The MET/AXL-induced epithelial-mesenchymal transition conferred resistance to sunitinib (5). Concomitant hyperactivation of the extracellular signal-regulated kinase (ERK)/signal transducer and activator of transcription 3 (STAT3) and rapamycin complex 2 (mTORC2)/AKT contributed to anti-apoptosis of metastatic RCC (6). So far, the clinical trials that attempted to conquer the anti-angiogenesis were limited, urging an extensive understanding of resistance mechanisms.

Mounting evidence has demonstrated that long non-coding RNAs (lncRNAs) were involved in tumor initiation, proliferation, metastasis, and recurrence. Many lncRNAs triggered abnormal activation of signaling cascades via interacting with chromatin, RNAs, or protein (7). Zhai et al., proved that the association of lncRNA-SARCC and androgen receptor hampered RCC progression by inhibiting AKT- and ERK-dependent pathways post-sunitinib treatment (8). Qu and colleagues revealed that lncRNA Activated in RCC with sunitinib resistance (lncARSR) promoted AXL and c-MET expression via sponging miR-34/miR-449, resulting in sunitinib resistance (9). Chen et al. found the expression of lncRNA LINC00461 and microRNA miR-942 was associated with poor clinical outcome of RCC (10). Although many lncRNAs have been identified in the exploration of novel therapeutic targets, the mechanisms of resistance against anti-angiogenic agents, especially for sunitinib, remained largely unknown.

In the present study, the authors validated that the lncRNA colon cancer-associated transcript-1 (CCAT1) increased in the sunitinib-resistant RCC and aimed to illustrate the mechanisms of CCAT1 and sunitinib resistance by conducting experiments *in vitro* and *in vivo*.

## Materials and Methods

### Patients and Specimens

Sixty patients with RCC, who received single sunitinib treatment pre- or postsurgery, were collected by the Department of Urology, Shengjing Hospital, China Medical University. The study was approved by the Ethics Committee of Shengjing Hospital, China Medical University. All participants provided written informed consent for research purposes and publication. The response to sunitinib, the histology subtypes, the expression of CCAT1, and the stages of the RCC cases are summarized in Table 1. All the specimens were kept in liquid nitrogen immediately.

**TABLE 1:** The correlation of lncRNA CCAT1 expression and stages of RCC.

Clinical features		Number of patients	CCAT1 expression		*χ* ^2^	*P*-values^a^
			
			Low	High		
Gender	Male	28	15	13	0.268	0.605
	Female	32	15	17		
Age (years)	<50	26	14	12	2.443	0.118
	≥50	34	11	23		
Depth of invasion (pT)	T1, T2	37	19	18	0.071	0.791
	T3, T4	23	11	12		
Lymph node metastasis (pN)	No	19	9	10	0.077	0.781
	Yes	41	19	22		
TNM stage	I	19	9	10	0.666	0.717
	II	18	10	8		
	III	23	12	11		
Histology	Papillary	19	9	10	0.372	0.542
	Clear	41	16	25		
Resistance to sunitinib	Sensitive	26	15	11	4.484	0.027*
		34	10	24		

*RCC*, renal cell carcinoma; *pT*, pathological primary tumor; *pN*, pathological regional lymph nodes. ^*a*^Fisher’s exact test with two-tailed *P*-value. *P < 0.05 indicated statistical significance.

### LncRNA Microarray Analysis

Total RNA from sunitinib-resistant sublines or the corresponding parental cells were used for nrStar^TM^ Human Functional LncRNA PCR Array (Arraystar Inc., Rockville, MD, United States). LncRNA microarray analysis was performed according to the manufacture’s instruction. LncRNAs (fold change >1.5, and *P*-value < 0.05) were considered expressed differentially between two groups. Each group was analyzed in triplicate. Data gained from three independent experiments were available in the Gene Expression Omnibus (accession No. GSE146305).

### Cell Culture and Establishment of Sunitinib-Resistant Sublines

Human immortalized normal kidney cell HK2, human papillary renal carcinoma cell (RCC) line ACHN, and clear RCC line 786-O were purchased from Cell Bank of the Chinese Academy of Sciences (Shanghai, China). Cells were cultured in Dulbecco’s modified Eagle’s medium (DMEM) (Gaithersburg, MD, United States) containing 10% fetal bovine serum (FBS) (Gaithersburg, MD, United States).

The parental ACHN and 786-O cells were treated with Sunitinib Malate (Selleck Chemicals, Houston, TX, United States) at an initial dose of 0.25 μM for 72 h. Subsequently, the concentrations of sunitinib increased by 0.25 μM every 72 h. The survival clones were subsequently passaged until the IC_50_ values reached 18 μM in the 10th month. The stable sunitinib-resistant sublines were maintained with the medium supplemented by 0.02 μM sunitinib.

### Plasmid Construction and Reagents

Colon cancer-associated transcript-1 was amplified and inserted into pLenti-P2A-Puro expression vector. The stably expressed CCAT1 subclones were selected and maintained with puromycin. sh-CCAT1 was synthesized and cloned into the HuSH pRS plasmid. The subsequent mammalian selection was accomplished with puromycin. All procedure was conducted following the manufacturer’s instruction (OriGene, Rockville, MD, United States). siRNA targeting c-Myc and the scrambled negative control were synthesized by OriGene as well. pcDNA3.3_c-Myc and the vector backbone pcDNA3.3 were gifts from Dr. Derrick Rossi (Addgene plasmid #26818; **RRID:** Addgene_26818)^1^ (11).

### MTT Assay

MTT assays were conducted to evaluate the cell viability, as previously described (12). In brief, cells were seeded at 1 × 10^4^/well in 96-well plates and were plated in 0.1 ml DMEM treated with different factors for 12, 24, 36, and 48 h. At each time point, 10 μl MTT solution (5 mg/ml) was added, followed by incubation for 4 h at 37°C. Then the medium was replaced by 150 μl dimethyl sulfoxide solution, followed by incubation for another 10 min to solubilize crystals. The optical densities were read at 490 nm using a Microplate Reader (Life Science, Hercules, CA, United States).

### Hoechst 33258 Staining

Hoechst 33258 staining dye solution (Abcam, Cambridge, United Kingdom) was used to detect the apoptosis of cells per the manufacturer’s protocols. The representative fluorescence photographs were shown.

### Colony Formation Assay

Cells at 1 × 10^4^ were seeded in 12-well Petri dishes. Twelve hours later, cells were treated with sunitinib for 48 h. Colony formation assays were conducted following the previously described instructions (13).

### Western Blot

Proteins were extracted using 100 μl lysis buffer form cells and tissues. Thirty micrograms of lysates were resolved with SDS-PAGE gel and transferred to nitrocellulose membranes through electroblotting. The membranes were blocked with 5% blocking solution for 1 h, followed by incubation with B-cell lymphoma 2 (Bcl-2; 12789-1-AP), myeloid cell leukemia 1 (Mcl-1; 16225-1-AP), c-Myc (10828-1-AP), and glyceraldehyde 3-phosphate dehydrogenase (GAPDH; 60004-1-Ig) antibodies (Proteintech Group, Inc., Rosemont, IL, United States) overnight at 4°C. The dilution of the primary antibodies was 1:1,000. Membranes were washed three times with TBST and incubated with HRP-conjugated secondary antibodies (Merck & Co., Inc., Kenilworth, NJ, United States) for another 1 h at a dilution of 1:2,500. Immunoreactivity was measured using the Western Lighting Ultra (Thermo Fisher Scientific, Waltham, MA, United States).

### Real-Time Quantitative Polymerase Chain Reaction

Total RNA was extracted by 1 ml TRIzol^TM^ Plus RNA Purification Kit (Thermo Fisher Scientific, Waltham, MA, United States) according to the manufacturer’s protocol. Then 200 ng RNA was reverse transcribed to cDNA in 20 μl system by SuperScript^TM^ reverse transcriptase kit (Thermo Fisher Scientific). Real-time PCR was performed using the Mx3000P real-time PCR system (Thermo Fisher Scientific). PCR was conducted as follows: 40 cycles of 94°C for 15 s, 60°C for 10 s, and 72°C for 20 s. All procedures were repeated thrice. Gene expression was normalized to the GAPDH to calculate relative expression using the 2^–Δ^ ^Δ^ ^*Cq*^ method (14). Primers used for qRT-PCR were synthesized by OriGene and listed in Table 2.

**TABLE 2:** Primers used in the reverse transcription-quantitative polymerase chain reaction.

Gene	Forward primer (5′-3′)	Reverse primer (5′-3′)
CCAT1	TTTATGCTTGAGCCTTGA	CTTGCCTGAAATACTTGC
GAPDH	GTCTCCTCTGACTTCAACAGCG	ACCACCCTGTTGCTGTAGCCAA
c-Myc	CCTGGTGCTCCATGAGGAGAC	CAGACTCTGACCTTTTGCCAGG
Bcl-2	ATCGCCCTGTGGATGACTGAGT	GCCAGGAGAAATCAAACAGAGGC
Mcl-1	CCAAGAAAGCTGCATCGAACCAT	CAGCACATTCCTGATGCCACCT

### Luciferase Reporter Assay

Dual-luciferase activity assays were performed as described previously (15). The pGL2-basic-Myc promoter was a gift from Dr. Linda Penn (Addgene plasmid #35156; **RRID**: Addgene_35156)^2^ (16). Twenty-four hours before transfection, 1 × 10^4^ cells were plated in a 96-well plate. sh-CCAT1 or CCAT1 was transfected into cells together with 60 ng of pGL2-basic-Myc promoter. Luciferase activity was determined with the dual-luciferase reporter assay system post-24 h transfection with the Luciferase Reporter Assay System (Promega, Madison, WI, United States).

### Animal Studies

All animal studies were approved by the Institutional Animal Care and Use Committee of Shengjing Hospital, China Medical University. Twenty-four 4-week-old female nude BALB/c mice (Vital River Laboratory Animal Technology Co., Ltd., Beijing, China) were housed in specific-pathogen-free conditions. Sunitinib-resistant ACHN subline (ACHN-SR) cells at 5 × 10^6^ introduced scrambled negative control RNA or small hairpin RNA (shRNA) targeting CCAT1 were subcutaneously injected into the flanks of mice. Mice with tumors were randomized into four groups (six mice per group) when the mean of tumor volume achieved 100–300 mm^3^. Each group received either saline or 20 mg/kg sunitinib by oral gavage daily for 28 days, according to the previous description (5). The tumor volumes were monitored every 4 days during the intervention. The mice were euthanized after scheduled treatment. Tumors were removed, weighed, and recorded followed the standard protocol.

### Statistical Analysis

The data from all experiments were presented as means plus standard deviation. The association between CCAT1 and c-Myc expression was analyzed by Pearson’s correlation coefficient. The differences were evaluated by one-way analysis of variance (ANOVA) with LSD test. *P* < 0.05 was considered statistically significant. Results represented the mean ± SD of three independently repeated experiments. Statistical analysis was conducted using GraphPad version 7.0 (San Diego, CA, United States).

## Results

### LncRNA CCAT1 Expression Escalates in Acquired Sunitinib-Resistant RCC

**FIGURE 1:**
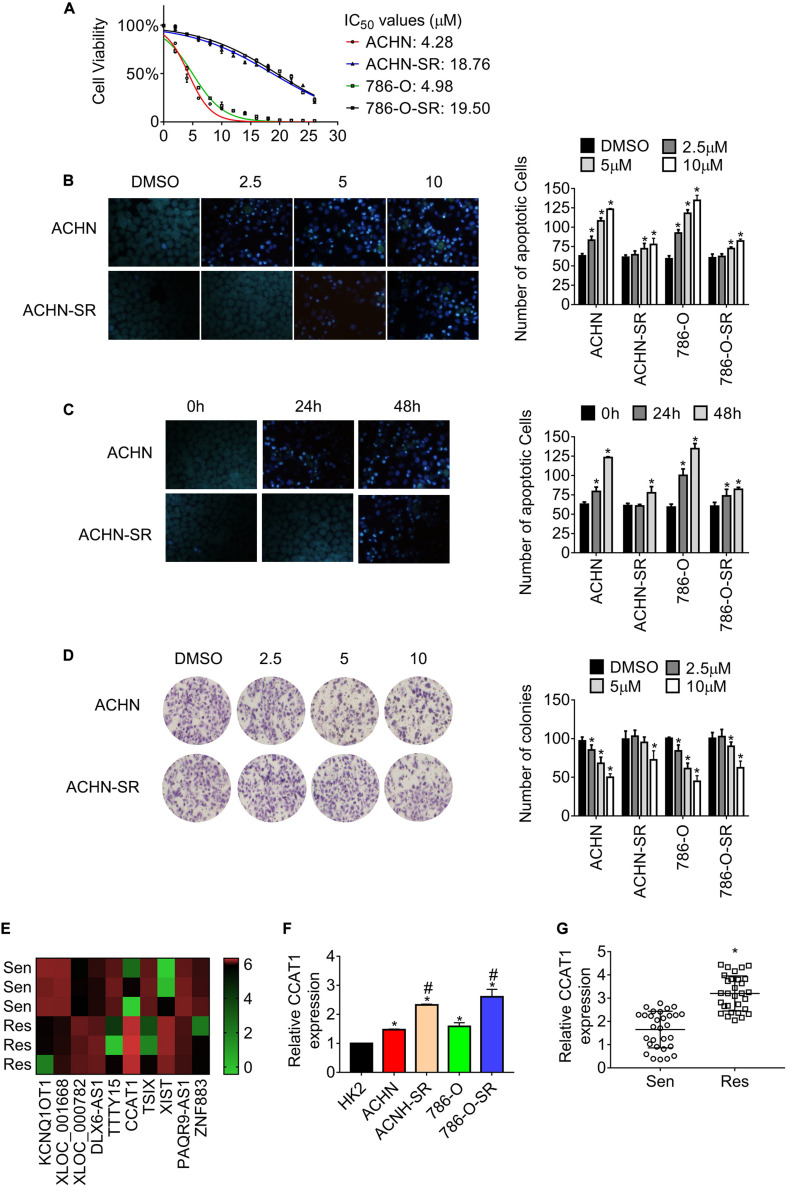
LncRNA CCAT1 expression increases in sunitinib-resistant RCC. **(A)** The half-maximal inhibitory concentration (IC_50_) of the sunitinib resistant and the corresponding parental cells were estimated by MTT assay. **(B)** The apoptosis of the indicated cells was measured by Hoechst 33258 staining. Cells were exposed to different doses of sunitinib for 24 h. *P < 0.05 vs. DMSO. **(C)** The apoptosis of the indicated cells was measured by Hoechst 33258 staining. Cells were exposed to 2.5 μM sunitinib before staining. *P < 0.05 vs. untreated cells. **(D)** The clonogenicity of the indicated cells was detected by colony formation assay. Cells were exposed to sunitinib prior to colony formation assay. *P < 0.05 vs. DMSO. **(E)** The heatmap showed the diverse expression of lncRNAs in the indicated cell lines. **(F)** The expression of lncRNA CCAT1 in the indicate cells was examined by RT-qPCR. *P < 0.05 vs. HK-2 cells. #*P* < 0.05 vs. parental cells. **(G)** The expression of lncRNA CCAT1 in RCC tissue that was sensitive or resistant to sunitinib. *P < 0.05 vs. sensitive samples. Results represented the mean ± SD of three independently repeated experiments. Sen, sensitive; Res, resistant. The represented images have a magnification of ×100.

According to the previous study (17), we established sunitinib-resistant sublines of ACHN and 786-O cells, named ACHN-SR and 786-O-SR, respectively. The half-maximal inhibitory concentration (IC_50_) for the resistant cells and the corresponding parental cells was determined by MTT assay. As shown in Figure 1A, the IC_50_ value of resistant cell lines was approximately fivefold those of parental cell lines. Moreover, the number of apoptotic SR cells reduced significantly compared with the parental cells when exposed to sunitinib (Figures 1B,C). Besides, the clonogenicity of SR cells remained consistent until cells were treated with 10 μM sunitinib, whereas the parental cells lost colony formation capacity postexposure with 2.5 μM sunitinib (Figure 1D). Similar results were gained in 786-O-SR and 786-O cells.

To investigate the differently expressed lncRNAs in SR sublines in comparison with the parental cells, we carried out lncRNAs microarrays and identified colon cancer-associated transcript 1 (CCAT1) as the most upregulated lncRNA (Figure 1E and Supplementary Table 1). The results in Figure 1F showed that CCAT1 expression intensified in SR cells compared with the relevant parental cells. Besides, the expression of CCAT1 increased in RCC cells in contrast with the immortalized normal kidney cell HK-2. Further examination revealed that CCAT1 expression raised in sunitinib-resistant RCC specimens compared with sunitinib-sensitive ones (Figure 1G).

Briefly, the evidence mentioned above proved that we generated acquired sunitinib-resistant RCC sublines and established the fact that lncRNA CCAT1 upregulated in sunitinib-resistant RCC.

### LncRNA CCAT1 Confers RCC Resistance to Sunitinib

**FIGURE 2:**
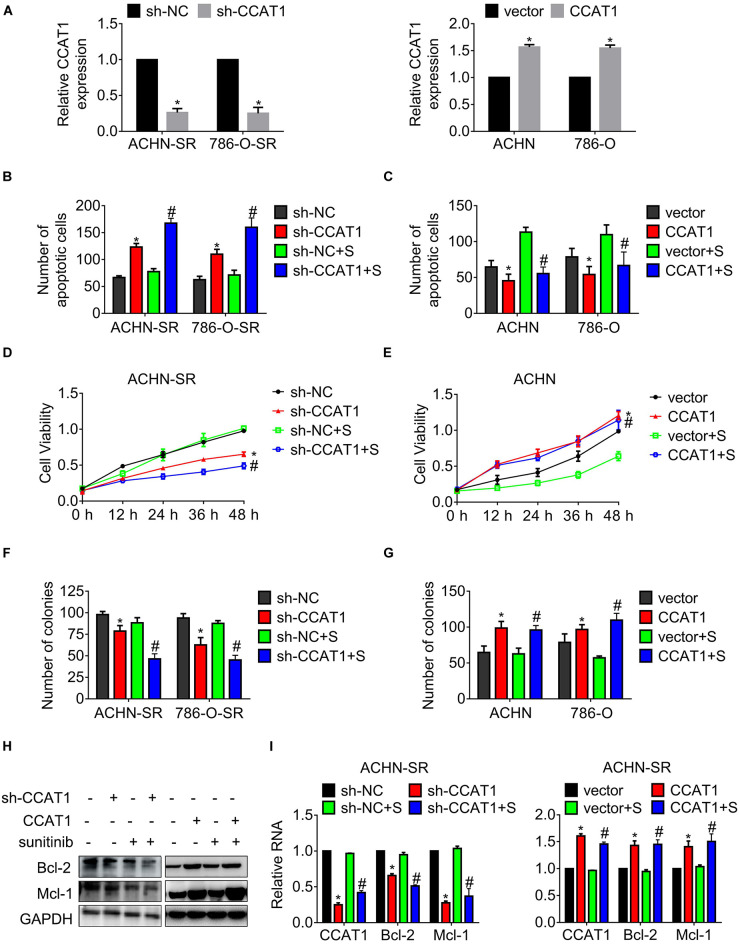
LncRNA CCAT1 confers resistance against sunitinib. **(A)** The expression of CCAT1 in cells expressing CCAT1 or sh-CCAT1 was detected by RT-qPCR. *P < 0.05 vs. vector or sh-NC. **(B,C)** The apoptosis of the indicated cells postexposure with sunitinib was measured by Hoechst 33258 staining. **(D,E)** The viability of the indicated cells post-exposure with sunitinib was accessed by MTT assay. **(F,G)** The clonogenicity of the indicated cells post-exposure with sunitinib was accessed by colony formation. **(H,I)** The expression of Bcl-2 and Mcl-1 in the indicated cells was accessed by western blot or qRT-PCR, respectively. *P < 0.05 vs. vector or sh-NC. #*P* < 0.05 vs. vector or sh-NC plus sunitinib. Results represented the mean ± SD of three independently repeated experiments. All cells were treated with 2.5 μM sunitinib. S, sunitinib.

To explore the association of CCAT1 expression and sunitinib resistance, we introduced short hairpin RNA targeting CCAT1 into SR cells, and CCAT1 mimics into parental cells, respectively. Figure 2A exhibits that CCAT1 declined in SR cells expressing sh-CCAT1 while that increased in parental cells expressing CCAT1. The results in Figure 2B showed that the deprivation of CCAT1 with shRNA triggered apoptosis of SR cells compared with the scrambled negative control. Moreover, the deprivation of CCAT1 enhanced the sunitinib-induced apoptosis. In contrast, overexpression of CCAT1 inhibited the apoptosis of parental cells in comparison with vector. Importantly, ectopic expression of CCAT1 antagonized the sunitinib-induced apoptosis (Figure 2C). The privation of CCAT1 exaggerated sunitinib-suppressed cell growth compared with the scramble negative control RNA (Figure 2D). In contrast, the CCAT1 overexpression reversed the sunitinib inhibition on cell viability (Figure 2E). The effects of CCAT1 on clonogenicity were accessed by colony assay as well. Figure 2F shows that sh-CCAT1 impeded the clonogenicity of SR cells and promoted the response to sunitinib. Contrariwise, CCAT1 augmented the colony formation and conferred resistance against sunitinib (Figure 2G).

Additionally, the expression of anti-apoptotic regulators Bcl-2 and Mcl-1 was examined by western blot and real-time quantitative polymerase chain reaction (RT-qPCR), separately. As shown in Figures 2H,I, the expression of Bcl-2 and Mcl-1 fell to a greater extent in SR cells deregulated CCAT1 post-sunitinib treatment compared with those post-DMSO.

These results indicated that CCAT1 played an essential role in driving resistance to sunitinib.

### Blockage of CCAT1 Conquers Resistance Against Sunitinib *in vivo*

**FIGURE 3:**
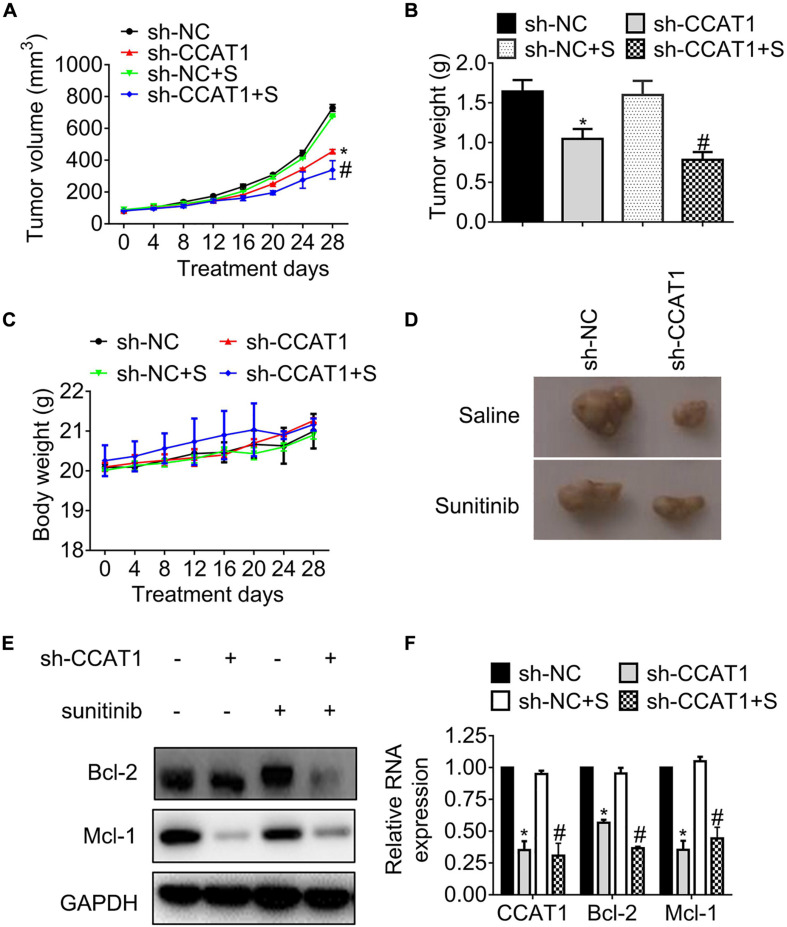
The knockdown of CCAT1 induces tumor regression in sunitinib-resistant ACHN mouse models. **(A)** The growth curves of tumors were recorded every 4 days. **(B)** The tumor weights were measured on day 28. **(C)** The body weights were recorded every 4 days. **(D)** The representative gross examination of tumors. **(E,F)** The expression of Bcl-2 and Mcl-1 was detected by western blot and qRT-PCR, respectively. *P < 0.05 vs. sh-NC. #*P* < 0.05 vs. sh-NC plus sunitinib. Results represented the mean ± SD of three independently repeated experiments. S, sunitinib.

To observe the effects of CCAT1 on tumor growth, we generated ACHN-SR mouse models by injecting ACHN-SR cells subcutaneously, followed by sunitinib intervention. The tumor growth curves shown in Figure 3A indicated that the deprivation of CCAT1 repressed tumor growth. Moreover, the impediment of CCAT1 promoted the sunitinib-induced tumor regression, whereas the sunitinib had little effect on the tumor growth of ACHN-SR. In line with the alterations of tumor growth curves, the tumor weights of sh-CCAT1 plus sunitinib reduced to the most extent in comparison with the sh-NC group (Figure 3B). Meanwhile, the body weights of xenografts varied hardly during the intervention, suggesting that the combination of sh-CCAT1 and sunitinib was tolerated (Figure 3C). Figure 3D shows the representative gross specimens of tumors, supporting the earlier results.

We further accessed the expression of apoptosis regulators Bcl-2 and Mcl-1. The results in Figures 3E,F demonstrated that the expression of Bcl-2 and Mcl-1 declined significantly, accompanied by the deregulation of CCAT1 in protein levels and RNA levels, respectively.

In brief, the hindrance of CCAT1 overcame resistance to sunitinib *in vivo*.

### CCAT1 Promotes c-Myc Expression in RCC

**FIGURE 4:**
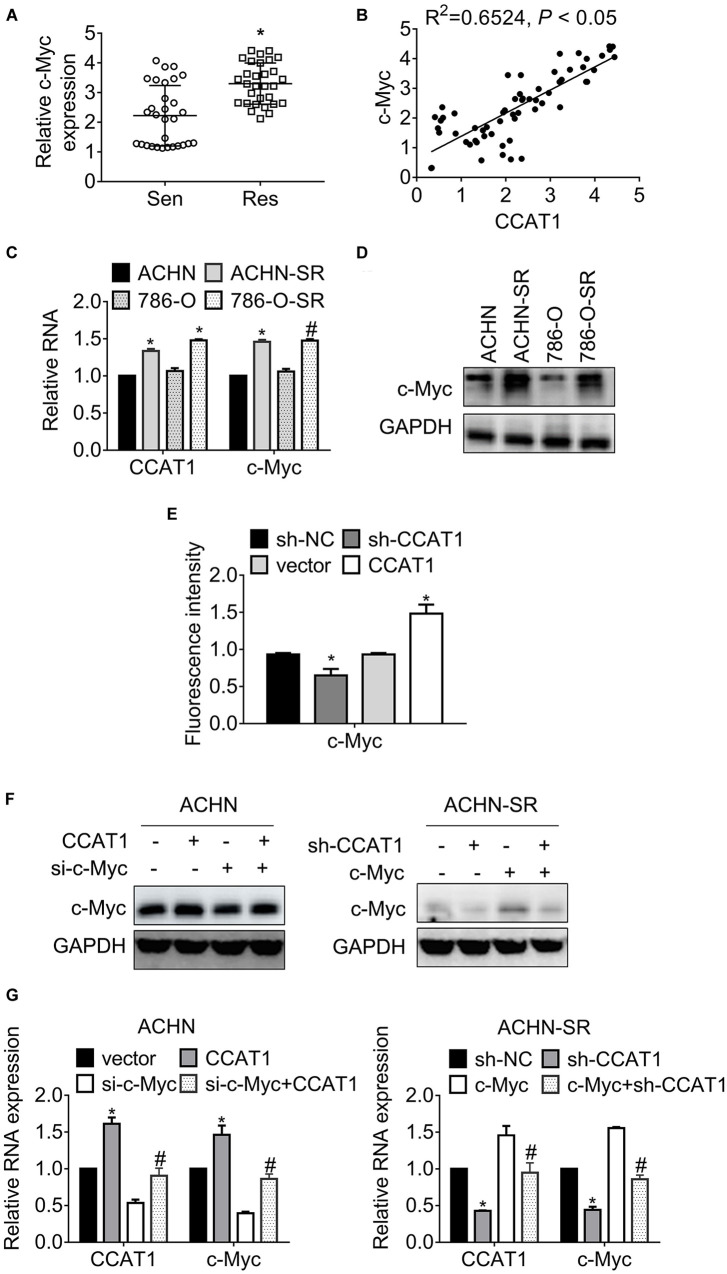
LncRNA CCAT1 promotes c-Myc expression. **(A)** The expression of c-Myc in sunitinib-sensitive or resistant RCC specimens were accessed by RT-qPCR. *P < 0.05 vs. sensitive groups. **(B)** The correlation of CCAT1 and c-Myc expression was estimated by Pearson’s correlation coefficient. *P* < 0.05 was considered statistical significance. **(C)** The expression of c-Myc in sunitinib-resistant and parental cell lines were accessed by western blot. *P < 0.05 vs. parental cells. **(D)** The expression of c-Myc in various RCC cell lines was accessed by western blot. *P < 0.05 vs. HK-2 cells. **(E)** The relative activity of *MYC* promoter was accessed by dual-luciferase assay. *P < 0.05 vs. vector or sh-NC. **(F)** The expression of c-Myc in the indicated cells were determined by western blot. *P < 0.05, vs. vector or sh-NC. **(G)** The expression of c-Myc in the indicated cells were determined by RT-qPCR. *P < 0.05 vs. vector or sh-NC. ^#^*P* < 0.05 vs. si-c-Myc or si-c-Myc. Results represented the mean ± SD of three independently repeated experiments.

Previous studies have evaluated that CCAT1 and c-Myc acted simultaneously in a positive feedback loop, provoking tumor progression (18, 19). We, therefore, attempted to illustrate the expression of c-Myc in 60 primary RCC specimens. The results in Figure 4A proved that c-Myc increased in sunitinib-resistant RCC samples compared with sunitinib-sensitive samples. Further analysis revealed that the expression of c-Myc and CCAT1 correlated significantly (Figure 4B). The association of c-Myc and CCAT1 contributed to approximately 65% of the alteration of c-Myc in the 60 RCC cases. Besides, c-Myc expression boosted in SR sublines compared with the parental cells (Figures 4C,D). The dual-luciferase assay validated that the activity of the c-Myc promoter fell by deregulation of CCAT1, while that augmented by overexpression of CCAT1 (Figure 4E).

To confirm the interaction between CCAT1 and c-Myc, we introduced CCAT1 plus c-Myc into SR sublines and parental RCC cells. Figure 4F presented that CCAT1 promoted c-Myc expression, and a combination of CCAT1 and si-c-Myc restored the c-Myc expression in ACHN cells. Conversely, sh-CCAT1 plus c-Myc reversed the c-Myc inhibition by sh-CCAT1. The alterations of CCAT1 and c-Myc at transcription levels agreed with the changes at translation levels (Figure 4G).

### CCAT1 Confers Sunitinib Resistance via Promoting c-Myc Expression

**FIGURE 5:**
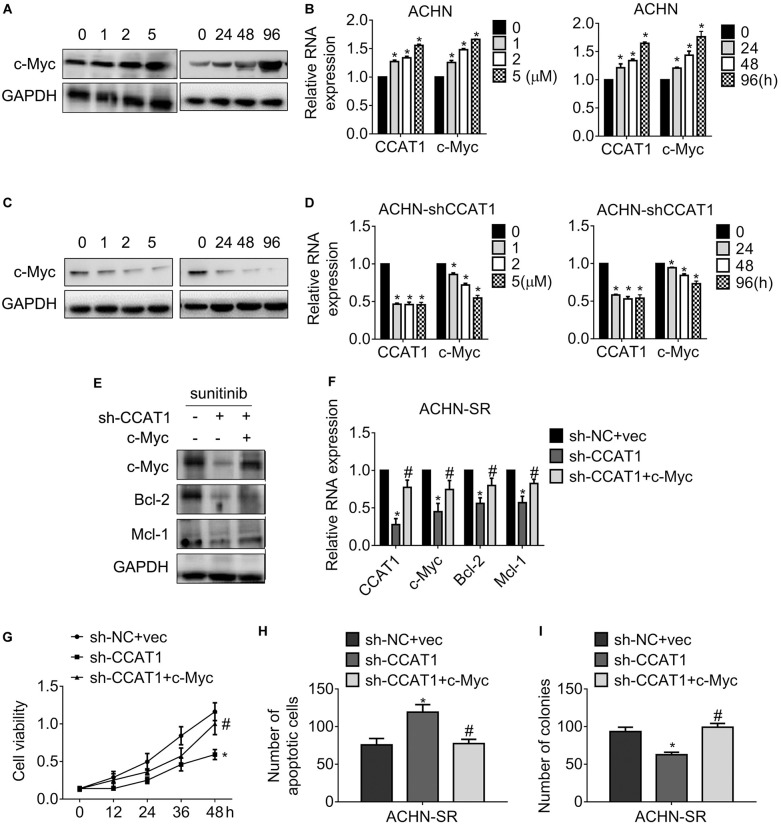
LncRNA CCAT1 drives sunitinib resistance in a c-Myc-dependent manner. **(A,B)** The expression of c-Myc in ACHN cells were accessed by western blot or qPCR, respectively. Cells were treated with different doses of sunitinib or 5 μM sunitinib for different durations. *P < 0.05 vs. control. **(C,D)** The expression of c-Myc in CCAT1-deprived ACHN cells were accessed by western blot or qPCR, respectively. Cells were treated with different doses of sunitinib or 5 μM sunitinib for different durations. *P < 0.05 vs. control. **(E,F)** The expression of the indicated genes was examined by western blot or RT-qPCR, respectively. Cells were introduced into sh-CCAT1 alone or sh-CCAT1 plus c-Myc before exposure with sunitinib. *P < 0.05 vs. sh-NC + vector; #*P* < 0.05 vs. sh-CCAT1. **(G)** The viability of the indicated cells was measured by MTT assay. **(H)** The apoptosis of the indicated cells was measured by Hoechst 33258 staining. **(I)** The clonogenicity of the indicated cells was measured by colony formation. *P < 0.05 vs. sh-NC + vector; #*P* < 0.05 vs. sh-CCAT1. Results represented the mean ± SD of three independently repeated experiments. Cells were exposed to 2.5 μM sunitinib apart from the indicated doses.

To study the relationship of CCAT1/c-Myc axis and the resistance against sunitinib, we exposed ACHN-SR cells to increasing doses of sunitinib for 24 h or 2.5 μM sunitinib for the various duration, followed by an examination of c-Myc expression. Figures 5A,B exhibited that c-Myc raised in sunitinib dose- and time-dependent manner with the presence of CCAT1. Inversely, the expression of c-Myc decreased post identical sunitinib treatment without CCAT1 (Figures 5C,D). Further investigation demonstrated that the concomitant introduction of sh-CCAT1 and c-Myc retrieved the expression of c-Myc, Bcl-2, and Mcl-1 after sunitinib exposure (Figures 5E,F). As expected, the combo of sh-CCAT1 and c-Myc contributed to ACHN-SR regained cell viability (Figure 5G), antagonization of apoptosis (Figure 5H), and clonogenicity (Figure 5I), leading to resistance to sunitinib. To sum up, CCAT1 conferred sunitinib resistance in a c-Myc-dependent manner.

## Discussion

Among various histological and molecular subtypes of RCC, clear cell RCC is the most frequent malignancy (20). The clear cell RCC was characterizing with mutated von Hippel–Lindau-hypoxia-inducible factor (VHL-HIF) signaling pathway, hyperactivated PI3K/AKT pathways, and *MOTR* mutation (21, 22). The mutant of the VHL gene activates hypoxia-inducible factor-2α (HIF-2α), subsequently triggered the transcription of the vascular endothelial growth factor (VEGF), platelet-derived growth factor-β (PDGF-β), and transforming growth factor-α (TGF-α). Surgical regimen remained the preferred intervention for localized RCC and locally advanced RCC, while the targeted therapy is used for treating metastatic RCC. The multitargeted tyrosine kinase inhibitor, sunitinib, achieved in improving the clinical outcomes by inhibiting VEGF and PDGF receptors (23). However, the broad emergence of resistance resulted in tumor progression eventually. The mechanisms underlying resistance to sunitinib were poorly understood.

LncRNA CCAT1 was amplified frequently in colorectal cancer and other cancers, including RCC, breast cancer, gliomas, lung cancer, osteosarcoma, and gastric cancer (24, 25). We found that CCAT1 expression increased in established sunitinib-resistant RCC sublines and acquired sunitinib-resistant clinical samples (Figure 1). We further uncovered that CCAT1 played a fundamental role in conferring resistance to sunitinib by conducting experiments *in vitro* and *in vivo* (Figures 2, 3). Two different histological subtypes of cell lines, clear cell RCC 786-O, and papillary RCC ACHN were used to conduct *in vitro* experiments. The similar results suggested that the CCAT1-mediated resistance to sunitinib did not depend on the clear cell subtype or the papillary subtype. Further investigation needs to clarify the relevance of the CCAT1/c-Myc axis and the histological subtypes in terms of sunitinib resistance.

In addition to CCAT1, lncARSR, LncRNA-SARCC, and LINC00461 were also involved in the regulation of sunitinib resistance in RCC (8–10). Besides, CCAT1 sponged miR-218, conquering gefitinib-caused apoptosis of lung cancer cells with EGFR mutant (26). CCAT1 overexpression contributed to chemoresistance in different tumors as well. For instance, CCAT1 triggered cisplatin resistance via sponging miR-130a-3p in lung cancer (27). Deregulation of CCAT1 impeded 5-FU resistance of colon cancer (28). The current evidence broadened the knowledge of lncRNAs and sunitinib resistance, suggesting that CCAT1 served as a pivotal oncogene in antagonizing targeted therapy.

A previous study has demonstrated that CCAT1 and c-Myc formed a double-positive feedback loop to promote the expression of each other (24). We, therefore, investigated the association of CCAT1 and c-Myc in sunitinib-resistant sublines. As expected, the expression of c-Myc correlated to CCAT1 positively in sunitinib-resistant RCC samples and sublines (Figure 4). Moreover, knockdown of CCAT1 attenuated c-Myc expression after chronic sunitinib exposure (Figure 5). There was an increasing amount of evidence to indicate that the CCATs family enhanced c-Myc expression via multiple mechanisms in solid cancer and leukemia. CCAT1 was located in an enhancer region and maintained the chromatin interaction between *MYC* and enhancers, promoting *MYC* transcription (29). Besides, CCAT1 sponged miRNAs let-7c, miR155-5p, let7b-5p, miR490-3p, and miR218-5p, modulating *MYC* expression indirectly (18, 30). Furthermore, another member of CCATs, CCAT2, promoted c-Myc expression through WNT signaling (31). The present study proved that the CCAT1/c-Myc axis participated in the regulation of resistance against sunitinib in a sunitinib dose-dependent manner, implying that the CCAT1/c-Myc signaling cascades were universal in various malignancies.

Elgendy et al. demonstrated that anti-apoptotic protein Bcl-2 and Mcl-1 increased accompanied by the activation of mTORC1 due to the tolerated sunitinib doses (32, 33). Hence, we examined the Bcl-2 and Mcl-1 expression in cells that deprived CCAT1 and ectopic-expressed c-Myc. Our observation proved that the downregulation of CCAT1 suppressed Bcl-2 and Mcl-1, whereas the introduction of c-Myc rehabilitated Bcl-2 and Mcl-1. The present findings were consistent with the previous studies. Another research also supported our observations. Maroto and colleagues evaluated that the double c-Myc/HIF-2α-positive staining indicated a lower progression-free survival of metastatic RCC despite single c-Myc-positive staining were related to poor prognosis without statistical significance (34). The study suggested that the potential crosstalk of CCAT1 and HIF pathways should be clarified further.

## Conclusion

In conclusion, this study identified CCAT1 as an oncogene in RCC metastasis, at least partly, by activating c-Myc-dependent anti-apoptosis and prosurvival. These results provide new insight into the role of CCAT1/c-Myc axis in sunitinib-resistant metastatic RCC and facilitate the development of novel agents to promote the response to sunitinib.

## Data Availability Statement

The original contributions presented in the study are publicly available. This data can be found here: the NCBI Gene Expression Omnibus (GSE146305).

## Ethics Statement

The studies involving human participants were reviewed and approved by the Ethics Committee of Shengjing Hospital, China Medical University. The patients/participants provided their written informed consent to participate in this study. The animal study was reviewed and approved by the Institutional Animal Care and Use Committee of Shengjing Hospital, China Medical University.

## Author Contributions

LS wrote the main manuscript. LS, WL, and YZ performed the experiments, data analysis, and contributed to the manuscript revisions. LS and YZ designed the research. All authors reviewed the manuscript, and read and approved the final manuscript.

## Conflict of Interest

The authors declare that the research was conducted in the absence of any commercial or financial relationships that could be construed as a potential conflict of interest.

## Footnotes

1
http://n2t.net/addgene:26818

2
http://n2t.net/addgene:35156
