# Corrigendum: Long non-coding RNA CCAT1 acts as an oncogene and promotes sunitinib resistance in renal cell carcinoma

**DOI:** 10.3389/fonc.2023.1247057

**Published:** 2023-08-23

**Authors:** Liping Shan, Wei Liu, Yunhong Zhan

**Affiliations:** ^1^ Department of Urology, Shengjing Hospital, China Medical University, Shenyang, China; ^2^ Emergency Department, First Hospital of China Medical University, Shenyang, China

**Keywords:** CCAT1, renal cell carcinoma, sunitinib, resistance, apoptosis

In the published article, there were errors in **Figures 2**, **4** and **5** as published. There seems to be overlaps in the images presented in the aforementioned **Figures 2H**, **4D**, **F**, **5A** and **E**. **Figures 2**, **4** and **5** and their caption appear below.

**Figure 2:**
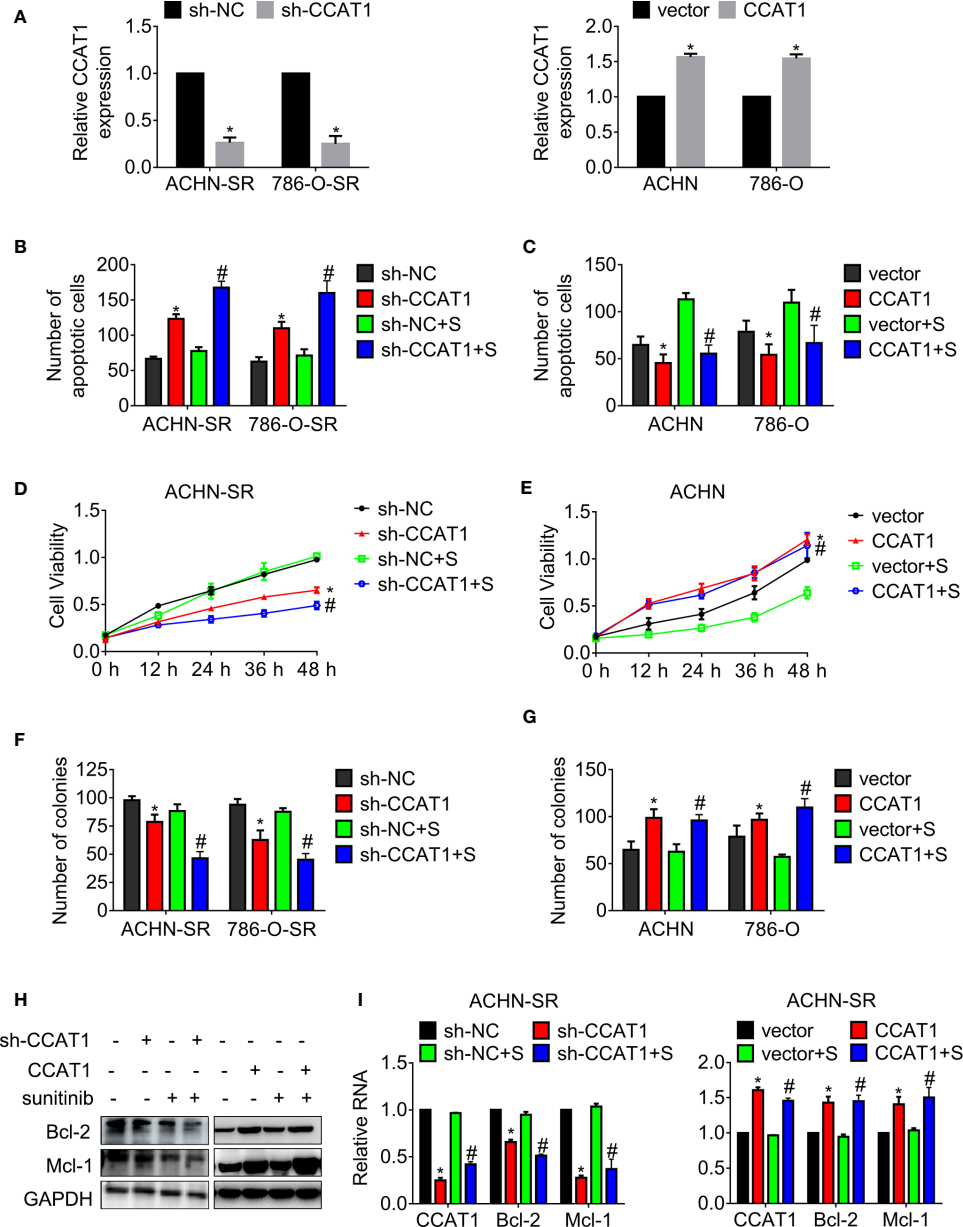
LncRNA CCAT1 confers resistance against sunitinib. **(A)** The expression of CCAT1 in cells expressing CCAT1 or sh-CCAT1 was detected by RT-qPCR. *P < 0.05 vs. vector or sh-NC. **(B, C)** The apoptosis of the indicated cells postexposure with sunitinib was measured by Hoechst 33258 staining. **(D, E)** The viability of the indicated cells post-exposure with sunitinib was accessed by MTT assay. **(F, G)** The clonogenicity of the indicated cells post-exposure with sunitinib was accessed by colony formation. **(H, I)** The expression of Bcl-2 and Mcl-1 in the indicated cells was accessed by western blot or qRT-PCR, respectively. *P < 0.05 vs. vector or sh-NC. #P < 0.05 vs. vector or sh-NC plus sunitinib. Results represented the mean ± SD of three independently repeated experiments. All cells were treated with 2.5 μM sunitinib. S, sunitinib.

**Figure 4:**
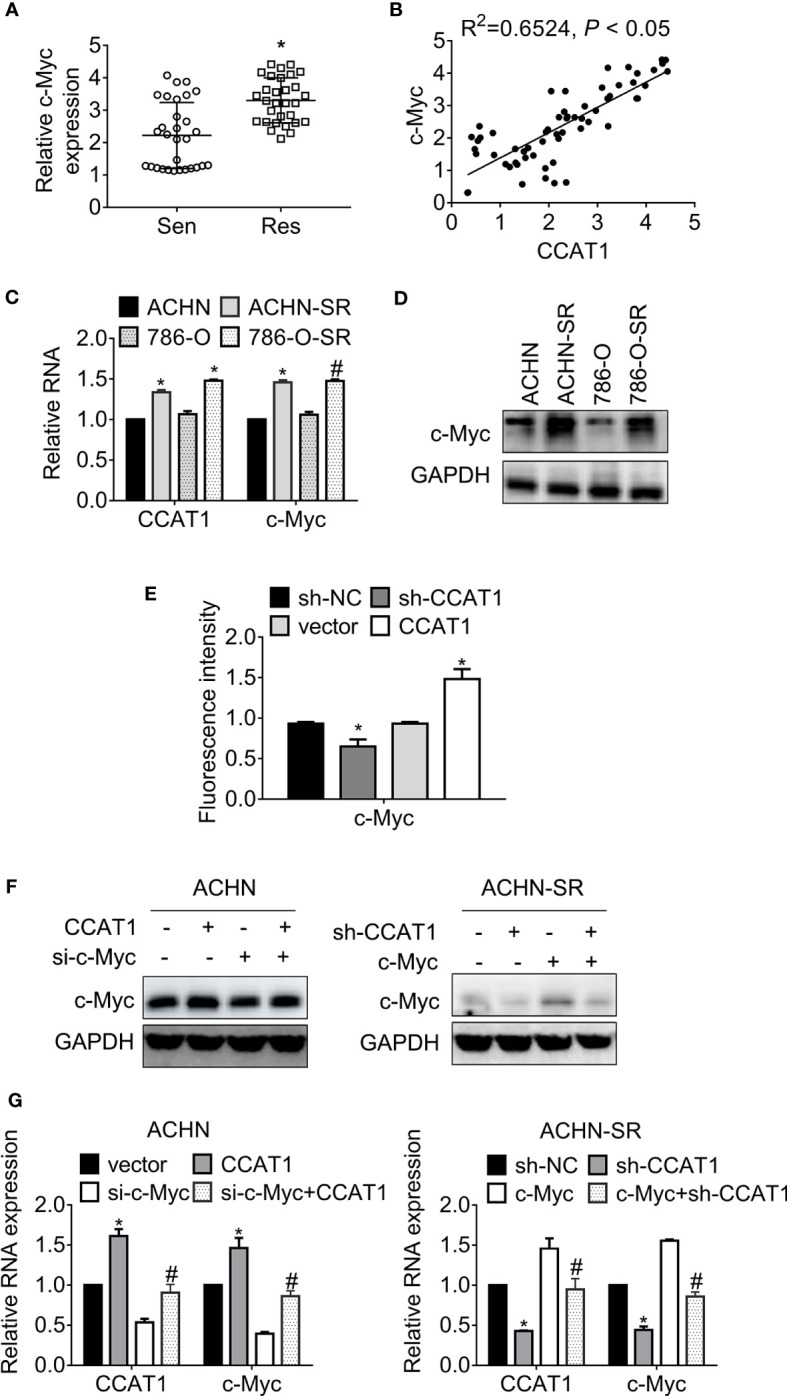
LncRNA CCAT1 promotes c-Myc expression. **(A)** The expression of c-Myc in sunitinib-sensitive or resistant RCC specimens were accessed by RT-qPCR. *P < 0.05 vs. sensitive groups. **(B)** The correlation of CCAT1 and c-Myc expression was estimated by Pearson’s correlation coefficient. P < 0.05 was considered statistical significance. **(C)** The expression of c-Myc in sunitinib-resistant and parental cell lines were accessed by western blot. *P < 0.05 vs. parental cells. **(D)** The expression of c-Myc in various RCC cell lines was accessed by western blot. *P < 0.05 vs. HK-2 cells. **(E)** The relative activity of MYC promoter was accessed by dual-luciferase assay. *P < 0.05 vs. vector or sh-NC. **(F)** The expression of c-Myc in the indicated cells were determined by western blot. *P < 0.05, vs. vector or sh-NC. **(G)** The expression of c-Myc in the indicated cells were determined by RT-qPCR. *P < 0.05 vs. vector or sh-NC. #P < 0.05 vs. si-c-Myc or si-c-Myc. Results represented the mean ± SD of three independently repeated experiments.

**Figure 5:**
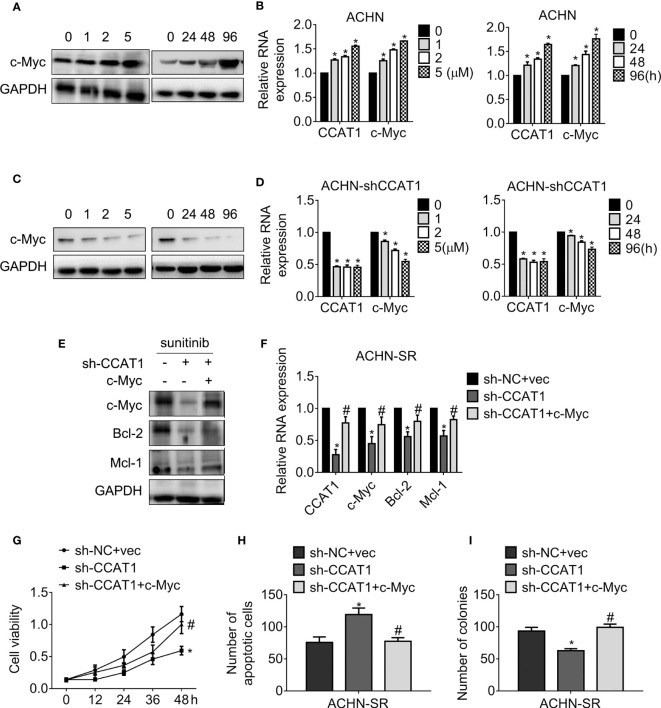
LncRNA CCAT1 drives sunitinib resistance in a c-Myc-dependent manner. **(A, B)** The expression of c-Myc in ACHN cells were accessed by western blot or qPCR, respectively. Cells were treated with different doses of sunitinib or 5 μM sunitinib for different durations. *P < 0.05 vs. control. **(C, D)** The expression of c-Myc in CCAT1-deprived ACHN cells were accessed by western blot or qPCR, respectively. Cells were treated with different doses of sunitinib or 5 μM sunitinib for different durations. *P < 0.05 vs. control. **(E, F)** The expression of the indicated genes was examined by western blot or RT-qPCR, respectively. Cells were introduced into sh-CCAT1 alone or sh-CCAT1 plus c-Myc before exposure with sunitinib. *P < 0.05 vs. sh-NC + vector; #P < 0.05 vs. sh-CCAT1. **(G)** The viability of the indicated cells was measured by MTT assay. **(H)** The apoptosis of the indicated cells was measured by Hoechst 33258 staining. **(I)** The clonogenicity of the indicated cells was measured by colony formation. *P < 0.05 vs. sh-NC + vector; #P < 0.05 vs. sh-CCAT1. Results represented the mean ± SD of three independently repeated experiments. Cells were exposed to 2.5 μM sunitinib apart from the indicated doses.

The authors apologize for this error and state that this does not change the scientific conclusions of the article in any way. The original article has been updated.

