# Supplementary material for: Long Non-coding RNA CCAT1 Acts as an Oncogene and Promotes Sunitinib Resistance in Renal Cell Carcinoma
Source: Front Oncol. 2020 Sep 25;10:516552. doi: 10.3389/fonc.2020.516552 (PMC7544819; doi:10.3389/fonc.2020.516552)
Supplement: Supplementary file 1 [file Table_1.DOCX]

**Supplementary Table**. Significantly expressed lncRNAs in RCC tissues.

| Gene name | trend | Fold change | *P* values |
| --- | --- | --- | --- |
| KCNQ1OT1 | Up | 14.077545 | 0.000129145 |
| XLOC_001668 | Up | 7.760521 | 0.00011794 |
| XLOC_000782 | Down | 0.153716 | 0.000219048 |
| DLX6-AS1 | Down | 0.283840 | 0.002900219 |
| TTTY15 | Up | 8.285778 | 0.000454544 |
| CCAT1 | Up | 63.602253 | 4.41912E-05 |
| TSIX | Up | 8.358793 | 0.000107489 |
| XIST | Down | 0.049284 | 0.0001138 |
| PAQR9-AS1 | Up | 2.586398 | 0.007375691 |
| ZNF883 | up | 3.970184 | 0.002845313 |
